# Genetic Gains in Pearl Millet in India: Insights Into Historic Breeding Strategies and Future Perspective

**DOI:** 10.3389/fpls.2021.645038

**Published:** 2021-03-30

**Authors:** Om Parkash Yadav, S. K. Gupta, Mahalingam Govindaraj, Rajan Sharma, Rajeev K. Varshney, Rakesh K. Srivastava, A. Rathore, Rajendra Singh Mahala

**Affiliations:** ^1^ICAR-Central Arid Zone Research Institute, Jodhpur, India; ^2^International Crops Research Institute for the Semi-Arid Tropics (ICRISAT), Patancheru, India; ^3^State Agricultural Biotechnology Centre, Centre for Crop and Food Innovation, Food Futures Institute, Murdoch University, Murdoch, WA, Australia; ^4^SeedWorks International Pvt Ltd, Hyderabad, India

**Keywords:** pearl millet, hybrid breeding, genetic gain, disease resistance, drought tolerance, biofortification, heat tolerance

## Abstract

Pearl millet (*Pennisetum glaucum* R. Br.) is an important staple and nutritious food crop in the semiarid and arid ecologies of South Asia (SA) and Sub-Saharan Africa (SSA). In view of climate change, depleting water resources, and widespread malnutrition, there is a need to accelerate the rate of genetic gains in pearl millet productivity. This review discusses past strategies and future approaches to accelerate genetic gains to meet future demand. Pearl millet breeding in India has historically evolved very comprehensively from open-pollinated varieties development to hybrid breeding. Availability of stable cytoplasmic male sterility system with adequate restorers and strategic use of genetic resources from India and SSA laid the strong foundation of hybrid breeding. Genetic and cytoplasmic diversification of hybrid parental lines, periodic replacement of hybrids, and breeding disease-resistant and stress-tolerant cultivars have been areas of very high priority. As a result, an annual yield increase of 4% has been realized in the last three decades. There is considerable scope to further accelerate the efforts on hybrid breeding for drought-prone areas in SA and SSA. Heterotic grouping of hybrid parental lines is essential to sustain long-term genetic gains. Time is now ripe for mainstreaming of the nutritional traits improvement in pearl millet breeding programs. New opportunities are emerging to improve the efficiency and precision of breeding. Development and application of high-throughput genomic tools, speed breeding, and precision phenotyping protocols need to be intensified to exploit a huge wealth of native genetic variation available in pearl millet to accelerate the genetic gains.

## Introduction

Pearl millet (*Pennisetum glaucum* R. Br.) is an important crop in the semiarid and arid ecologies of South Asia (SA) and sub-Saharan Africa (SSA) that are characteristically challenged by low and erratic rainfall and high mean temperature and simultaneously have soils with low organic carbon and poor water-holding capacity (Serba et al., 2020). Besides its unmatchable capacity to tolerate drought, pearl millet has also built-in adaptation to soils with low fertility. Because of its remarkable ability to respond to favorable environments because of its short developmental stages, high photosynthetic efficiency, and abundant capacity for high growth rate, pearl millet is an excellent crop for the short growing season and under improved crop management (Yadav and Rai, 2013) and is emerging as an important alternative crop for feed, food, fodder, and relay crop in Brazil, Canada, Mexico, United States, West Asia and North Africa region, and Central Asia.

Pearl millet is valued for its nutrient-rich grain for human consumption and its green fodder and dry stover for livestock (Andrews and Kumar, 1992) and forms the basis of livelihood and nutritional security for more than 90 million people in SSA and SA (Serba et al., 2020). Pearl millet demand is anticipated to increase in the future because of increasing human and livestock populations in SSA and SA and as a healthy food and other industrial uses (Rai et al., 2008). Its cultivation may further extend in the areas where maize and sorghum are cultivated because of depleting water resources. Pearl millet production is likely to become more challenging because of predicted intense drought stress, rise in temperature, and greater disease incidences in SSA (Sultan et al., 2013) and SA (Rama Rao et al., 2019). Therefore, its production must be increased at a much faster rate and more so in challenging agroecologies. Increasing production must come through enhancement in productivity as there is little scope to enhance production by expanding its cultivation, especially in SA.

Enormous progress has been made in India to improve productivity by developing high-yielding cultivars and their improved agronomic management during the last six decades (Jukanti et al., 2016). The accomplishments of pearl millet breeding are often referred to as one of the greatest success stories in Indian agriculture (Yadav et al., 2019). However, the biological potential of pearl millet has not been fully realized as indicated by the current 1.2 ton/ha national productivity in comparison to the productivity level of 4–5 tons/ha in the summer season in northwestern India. An attempt is made here to critically analyze past breeding strategies followed in pearl millet. We also examine the prospects of further accelerating genetic gains to meet the greater demand for pearl millet and to make its cultivation more profitable.

## Historic Breeding Strategies

Breeding strategies in pearl millet have evolved very comprehensively over several decades, taking into account understanding of its pollination behavior, challenges in its production, access to germplasm, and accumulated knowledge in the fields of its genetics, physiology, pathology, and so on.

Pearl millet is a highly cross-pollinated species, with outcrossing rates of more than 85%, because of its protogynous nature of flowering. Therefore, individual plants of natural populations mate randomly and are highly heterozygous and heterogeneous. Early breeding efforts in genetic improvement of pearl millet, which started as early as the 1930s, attempted to capitalize on such existing genetic variation within traditional landraces by subjecting them to simple mass selection (Athwal, 1961). The greater urgency for population improvement programs started with the acquisition of a diverse range of germplasm from across the world in the 1970s with the establishment of the International Crops Research Institute for the Semi-Arid Tropics (ICRISAT) (Gill, 1991; Witcombe, 1999). Eventually, a large number of populations and trait-based composites of the broad genetic base were established, and a diverse range of elite breeding materials was developed (Rai and Anand Kumar, 1994; Rai et al., 2006).

Like maize, the heterosis was also observed in pearl millet for grain yield (Athwal, 1966), but the hermaphrodite nature of flowers of small size limited the ability to exploit it at the commercial level. There were some innovative attempts in the 1950s to exploit heterosis through developing “chance hybrids” that included growing two parental populations of similar maturity in the mixture and allowing them to cross-pollinate to produce seed that contained ~40% hybrid seed (Gill, 1991). The chance hybrids outyielded local varieties by 10–15% but could not become popular because of lack of efficient seed production programs and their limited genetic superiority.

The discovery of cytoplasmic male sterility (CMS) in 1958 (Burton, 1965) at Tifton, Georgia, and the availability of good fertility restorers in Indian germplasm led to the development of commercial hybrids, making a quick and significant impact on pearl millet production (Gill, 1991). However, the cultivation of a limited number of hybrids over several years led to a downy mildew (DM) epidemic in the early 1970s (Safeeulla, 1976). Such epidemics reappeared whenever a few hybrids occupied a large area year after year (Singh, 1995). No association of A1 cytoplasm was established with the DM epidemic (Yadav et al., 1993; Yadav, 1996).

Recurring DM epidemics in pearl millet hybrids in India prompted to intensify efforts on genetic diversification of hybrid parental lines, especially after the 1980s. This involved both cytoplasmic and nuclear diversification of parental lines. In addition to A1 CMS source (Burton, 1965), A2, A3 (Athwal, 1961, 1966), A4 (Hanna, 1989), and A5 sources (Rai, 1995) were reported. Extensive characterization of these sources established instability of A2 and A3 sources, whereas A4 and A5 were found as more promising (Rai et al., 1996). This was followed by the development and dissemination of 89 A lines based on A4 and 27 based on A5 source by ICRISAT, but utilization of these two sources remained restricted because of lack of suitable restorers. ICRISAT initiated breeding efforts for developing restorers, especially for the A5 CMS system (Rai et al., 1996, 2009b). Research programs in India have now started breeding both A and R lines and developing hybrids based on these CMS systems. The understanding of the genetics of A4 (Gupta et al., 2012a) and A5 CMS (Gupta et al., 2018) helped in the well-organized and efficient utilization of these CMS sources.

A range of germplasm material from India and Africa with diverse phenotypic characteristics, such as tillering, panicle size, earliness, grain size, grain color, and so on, was strategically exploited to diversify the genetic base of both seed and restorer parents (Andrews and Anand Kumar, 1996; Rai et al., 2009a; Yadav et al., 2012c; Patil et al., 2020). In the last four decades, hybrid breeding has received a very high priority in India using genetically diverse parental lines targeting various production ecologies that have helped to intensify the genetic gains (Rai et al., 2009a; Yadav et al., 2012a).

## Trait Prioritization

Pearl millet is cultivated under diverse agroecologies ranging from near-optimum environments (with high use of irrigation and chemical fertilizers) to extremely challenging drought-prone environments (with little external inputs). This led to the prioritization of research to improve necessary phenotypic traits, climate adaption, disease resistance, and nutritional traits taking full cognizance of production constraints and differential requirements of various regions.

### Phenotypic and Productivity-Related Traits and Their Linkage With Megaenvironments

A single pearl millet cultivar cannot be expected to perform well under all the environmental conditions, and a cultivar planted outside its adaptation zone would suffer yield reduction due to significant genotype × environment interactions. Therefore, breeding and evaluation require a subdivision of the testing environments into relatively more homogeneous groups of locations, called megaenvironments, where specific genotypes can be targeted for individual megaenvironment.

**Figure 1:**
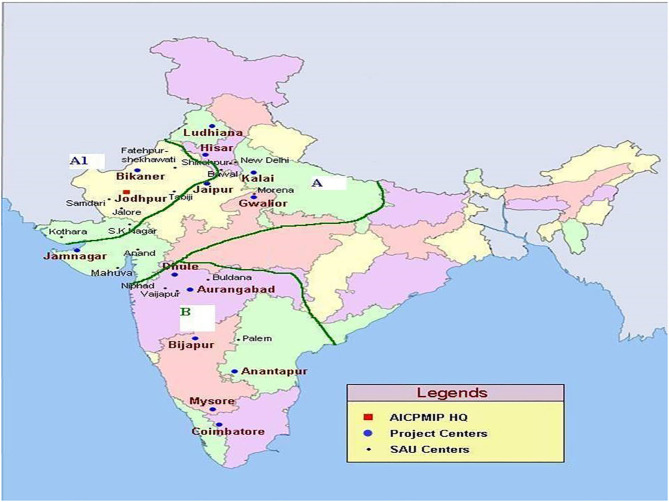
Three mega-environments (designated as A1, A- and B- zones) of pearl millet cultivation in India.

Indian pearl millet cultivation area has been divided into three megaenvironments (designated as A_1_, A, and B zones) considering the geographical location, rainfall pattern, local adaptation, and other environmental conditions (Gupta et al., 2013). The A zone consists of parts of northern India receiving >400 mm of annual rainfall (Figure 1). The A1 zone consists of parts of northwestern India receiving <400 mm of annual rainfall, whereas the B zone accounts for the area in peninsular India receiving more than >400-mm annual rainfall. At present, ~75% of the pearl millet is grown in A and A1 zones and 25% in B zone. Different pearl millet breeding programs in India have developed their product profiles, depending on the need of their target megaenvironment. ICRISAT, as a CGIAR center, which has a global mandate, is targeting all the megaenvironments to support national breeding programs.

High grain yield, disease resistance, and maturity duration of 75–85 days, as per the agroecological requirements, have been accorded the highest priority (Yadav and Rai, 2013). Because of the growing importance of dry stover for fodder purposes, there has been a considerable emphasis, in recent times, on breeding for dual-purpose cultivars producing both high stover and grain yields (Yadav et al., 2012b). Some of the traits in hybrids common to all the environments include lodging resistance, compact panicles, good exertion, and good seed set.

**Figure 2:**
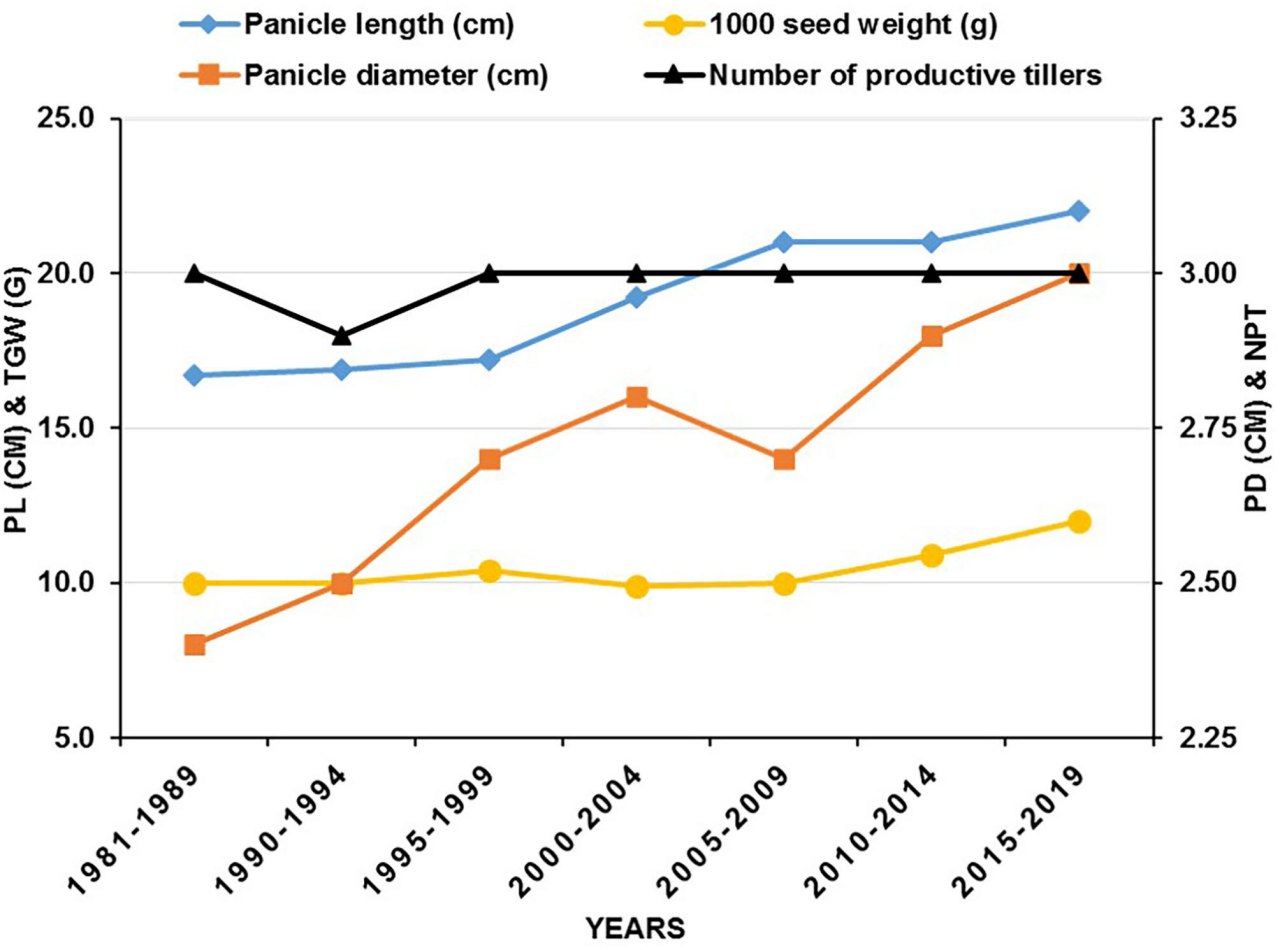
Comparison of different traits in seed parents (A/B-lines) developed between 1981 and 2019 at ICRISAT, Patancheru (PL, panicle length; TGW, thousand grains weight; PD, panicle diameter; NPT, number of productive tillers per plant).

Traits that have regional preferences include various maturity types, tillering, panicle size (a combination of panicle length and girth), and seed size and have been strategically manipulated. Genetic variation for these traits is widely available with high heritability (Yadav et al., 2004, 2017), and simple selection has been, therefore, very successful (Rattunde et al., 1989). Panicle length has increased from 16.7 to 22.0 cm, panicle diameter from 2.4 to 3.0 cm, and 1,000-grain weight from 10 to 12 g in seed parents developed during the last four decades (Figure 2). However, the mean productive tillers per plant were found unchanged.

The d_2_ dwarfing gene-based shorter height is the most dominant plant type developed in seed parents breeding (Rai and Hanna, 1990; Rai and Rao, 1991) as it reduces the risk of lodging in high-management conditions and helps in easy detection of off-type and pollen shedders in the seed production plots.

The A lines have been bred for complete and stable male sterility and B lines for profuse pollen production ability across the seasons and sites. In the breeding of A lines, high grain yield potential, both as lines *per se* and in hybrids (i.e., combining ability), is the most important consideration. High yield, however, is achieved in combination with other agronomic and farmers' preferred traits. The foremost requirement in the restorer lines is to produce highly fertile hybrids and to produce profuse pollen that remains viable at air temperatures as high as 42–44°C. It is desirable to breed pollinators that are taller than A lines, usually in the range of 150–180-cm height.

There has been a clear distinction between the public and private sector hybrid breeding programs regarding trait-based breeding. For instance, private sector breeding programs have largely focused on relatively better-endowed environments (A and B zones), giving greater emphasis to breeding dual-purpose hybrids. As a result, private sector hybrids are generally taller, later in maturity, with longer panicles and less number of effective tillers/plant [All India Coordinated Research Project on Pearl Millet (AICPMIP), unpublished data] as these traits are reported to contribute toward higher productivity in better-endowed environments. In contrast, most of the public sector hybrids are generally shorter in height and early in maturity, with smaller panicles and a higher number of effective tillers/plant. The private sector has also placed greater emphasis on breeding high grain yield and large-seeded hybrids.

The strategy of trait diversification led to the development of a diverse array of high-yielding hybrids with adequate adaptation to different agroecologies. This was evidenced from a recent investigation where 122 commercial hybrids showed the existence of significant variation for flowering time (42–58 days), tillering (1.1–4.4 panicles/plant), individual grain size (7.6–17.3 mg), plant height (185–268 cm), and panicle length (20–33 cm), which highlighted the successful efforts of the national program of pearl millet improvement toward genetic diversification of hybrids (Yadav et al., 2017).

### Climate Adaptation

Pearl millet is challenged by drought and heat stress in various production environments of SA and SSA. While drought is of common occurrence, heat stress assumes importance in specific regions.

#### Drought Tolerance

Drought, caused by the low rainfall and its erratic distribution, is the primary abiotic production constraint in SA and SSA. Efforts have therefore been made for mapping and delineation of drought-prone regions to define the target population of environments or megaenvironments (Gupta et al., 2013), which are highly variable in terms of their timing, intensity, and duration of drought (van Oosterom et al., 1995).

The subject of drought tolerance in pearl millet has remained, so far, a strategic research issue, and therefore, the response to drought has been studied comprehensively. Drought during the seedling stage results in poor plant stands reducing yield severely (Soman et al., 1987). Drought during the vegetative stage of growth has a little adverse effect on productivity (Bidinger et al., 1987) as there is a significant increase in panicle number, which is a reflection of a compensation mechanism for a damaged main shoot during drought (van Oosterom et al., 2003). However, the grain-filling stage is the most sensitive growth stage to drought as both grain number and grain size are significantly reduced when the crop is exposed to drought stress at this stage (Fussell et al., 1991).

Dissection of drought tolerance in terms of physiology, phenology, and morphology of the crop has led to the understanding of the yield formation process under drought (van Oosterom et al., 2003; Yadav, 2011), helping breeders to identify and target specific traits in different drought environments. Use of physiological traits as selection criteria for drought tolerance has been very challenging especially when dealing with a large number of genotypes in breeding nurseries. Drought escape mechanism has been successfully exploited by targeting early maturity for getting greater genetic gains in the drought-prone regions of northwestern India (Yadav et al., 2011). Morphological traits such as high tillering, small grain size, and shorter grain filling periods that can be measured easily have been manipulated successfully in breeding programs as there is an abundant variation available for these traits (Yadav et al., 2017).

The role of adapted germplasm has also been emphasized for drought breeding as the measured performance under drought stress is largely a result of adaptation to stress conditions (Yadav et al., 2009, 2012c). Hybridization of adapted landraces with elite genetic material creates new gene combinations that lead to amalgamating of adaptation to stress environments and high productivity (Presterl and Weltzien, 2003; Yadav and Rai, 2011, Patil et al., 2020). Genome regions underlying drought tolerance-related traits have been identified and mapped (Yadav et al., 2002, 2004; Serba and Yadav, 2016). Several such genomic regions are being manipulated to enhance drought tolerance (Bidinger et al., 2007; Sharma et al., 2014).

#### Heat Tolerance

Optimum temperature for normal growth of pearl millet is 33/34°C. Higher temperatures can affect the pearl millet both at the seedling and reproductive stages. Climate change models project that the pearl millet yield in SSA and SA will decrease by 6–17% by 2050 (Knox et al., 2011).

In India and western and southern Africa, soil surface temperatures often exceed 45°C and may sometimes reach 60°C and are one of the most important factors causing poor plant stands as pearl millet seedlings are most vulnerable to high temperatures during their first 10 days (Soman et al., 1981; Peacock et al., 1993). Therefore, improvement in heat tolerance at the seedling stage assumes importance. Genetic differences in seedling survival under high soil surface temperatures have been reported (Peacock et al., 1993), and selection for greater seedling emergence under artificial screening technique (Soman and Peacock, 1985) has been found effective (Lynch, 1994). A laboratory method based on measuring membrane thermostability has been developed (Howarth et al., 1997) to assess the differences in seedling heat tolerance. There has been no report of manipulating this trait in breeding programs either in SA or SSA in the last three decades.

During the last decade, pearl millet has emerged as a highly productive and remunerative crop in the hot and dry summer season in the northern and western parts of India (Yadav and Rai, 2013). With higher air temperatures (often >42°C) coinciding with flowering in this season, the crop suffers from reproductive sterility, leading to drastic reductions in seed set and finally into lesser grain yield (Gupta et al., 2015b; Djanaguiraman et al., 2018). Heat tolerance as the reproductive stage has emerged as an important target trait to enhance genetic gains.

Flowering-period heat stress screening protocols have been standardized for screening under both controlled environment facilities (growth chambers) and field conditions in heat stress–prone target ecology (Gupta et al., 2015b). Multilocational and multiyear field screening in summer season involving a large number of hybrid parental lines, germplasm accessions, and improved populations established that stigma is more heat-sensitive than pollen; large genetic variation exists between breeding lines and within open-pollinating populations; the boot-leaf stage is more heat-sensitive than panicle-emergence stage, and heat tolerance behaves as a dominant trait. These screenings led to the identification of heat-tolerant breeding materials that have been used to further enhance heat tolerance (Gupta et al., 2016, 2019) and facilitated the pyramiding of heat tolerance in high-yielding hybrids.

### Biotic Stress Resistance

Pearl millet is a hardy crop vulnerable to only fewer diseases and insect-pests compared to other major cereals. However, the diseases that attack pearl millet are capable of causing huge damage. Being a crop grown by resource-poor framers, diseases and pests are best managed through host plant resistance (HPR) as it does not incur an additional cost.

#### Diseases

Disease management through HPR involves a sound knowledge of biology and epidemiology of diseases, availability of pure culture of the pathogens, effective inoculation techniques, greenhouse and field screening facilities, appropriate disease rating scales, and availability of resistance sources.

##### Downy Mildew

DM caused by *Sclerospora graminicola* (Sacc.) J. Schröt is the most important disease causing heavy economic losses in India and Africa. Greenhouse and field screening of a large number of germplasm accessions and breeding lines has led to the identification of several resistance sources (Singh S. D. et al., 1987; Singh et al., 1997), which have been extensively used to develop DM resistant hybrids. The diversified genetic base of hybrids has contributed to the control of widespread DM epidemics.

Regular monitoring of changes in the virulence of pathogens and resistance of hybrids has helped in keeping track of the breakdown of resistance in hybrids. Replacing the susceptible hybrid with its disease-resistant version, created by marker-assisted selection, has also been an effective strategy (Hash et al., 2006).

##### Blast

The blast or leaf spot disease, caused by *Pyricularia grisea* Sacc. [syn. *Magnaporthe grisea*], has emerged as a very destructive disease of pearl millet in the recent past (Rai et al., 2012). Monitoring of virulence of pathogen populations and screening of genetic resources led to the identification of stable resistant lines to develop blast-resistant hybrid parent lines (Sharma et al., 2013; Yella Goud et al., 2016).

Resistance in pearl millet to Indian isolates of *M. grisea* is governed by a single dominant gene (Gupta et al., 2012b; Singh et al., 2018b), which makes the incorporation of resistance much easier. Molecular markers are also being used to identify Quantitative Trait Loci (QTLs) for blast resistance against prevalent pathotypes. Two major blast resistance QTLs, on LG 4 and LG 7 of pearl millet line 863B-P2, were identified that have been used to improve hybrid parental lines.

##### Rust

Rust (*Puccinia substriata* var. *indica* Ramachar & Cumm) is generally considered as a disease of less importance in the grain crop; however, it is of great importance in fodder crop where it reduces both the quantity and quality of the produce. The field screening in the late rainy season under high disease pressure and greenhouse screening led to the identification of stable resistance sources (Singh S. D. et al., 1987; Singh et al., 1990; Sharma R. et al., 2020).

##### Smut and Ergot

Ergot (*Claviceps fusiformis* Lov.) and smut (*Moesziomyces penicillariae* Bref. Vanky) are important floral diseases, and grain yield losses are proportional to their severity. Both pathogens are soil-borne and infect the host at flowering through stigma. Pollination before pathogen infection prevents infection (Thakur and Williams, 1980). These diseases are more severe in wet weather primarily because of pollen wash. The higher susceptibility of hybrids compared to the open-pollinated varieties is attributed to their greater uniformity in flowering time, rather than their cytoplasm (Yadav et al., 1992; Rai and Thakur, 1996).

Understanding the biology and epidemiology of these diseases helped the development of field screening techniques (Thakur and Rai, 2002). A large number of lines have been evaluated for their reaction to these diseases (Thakur and Rai, 2002; Abraham et al., 2019). Very high levels of ergot resistance in the germplasm accessions were not observed; hence, ergot-resistant lines were developed by intermating less susceptible plants and selecting and rescreening resistant progenies for several generations under high disease pressure (Thakur et al., 1993).

Smut resistance is a dominant trait and easily transferable. However, quantitative resistance involving additive and non-additive gene effects has also been reported (Thakur et al., 2011). A large number of lines have been found as resistant to smut and DM (Thakur et al., 1992, 2011). Currently, multilocational testing across seasons to evaluate the severity of smut and ergot on new experimental cultivars is being done to ensure no smut or ergot susceptible cultivar is released for cultivation.

#### Insect-Pests

More than 100 insect-pests have been reported to be associated with the pearl millet–based cropping system, but only a few of them are potential pests of any significant economic importance. These are shoot fly (*Atherigona approximata*), stem borers (*Chilo partelus* in India and *Coniesta ignefusalies* in western Africa) and white grubs (*Holotrichia consanguinea*) in India, earhead worms (*Helicoverpa armigera*), gray weevil (*Myllocerus* species), and leaf roller (*Marasmia trapezalis*) (Raghvani et al., 2008). Nature of damage and life cycle of these pests have been studied, and control measures developed. The distribution and damage of insect-pests vary in different regions. Long-term monitoring revealed that no single method of control is effective against any insect. It requires an integrated approach including cultural and chemical control (Sharma and Youm, 1999). The insect-pest incidence on commercial cultivars and experimental test genotypes is closely monitored, and no breeding programs are undertaking insect resistance as target trait.

### Grain Nutrition

The goal of core breeding has been to increase yield potential in view that pearl millet has been considered as a highly nutritious cereal with higher levels of proteins and several minerals than other major cereals (Singh and Nainawatee, 1999). Earlier research reported up to 24.3% protein content in germplasm (Jambunathan and Subramanian, 1988) and up to 19.8% in elite breeding lines (Singh P. et al., 1987). However, no serious efforts were made to improve protein content because of its negative correlations with grain yield (Singh and Nainawatee, 1999). Improving grain nutritional traits is a recent addition of breeding objective, in view of global recognition of widespread deficiencies of iron (Fe) and zinc (Zn). The major areas addressed include the assessment of the extent of genetic variation for grain Fe and Zn contents, identification of diverse seed-mineral dense germplasm, nature of genotype × environment interaction, relationships between grain minerals and agronomic traits, and genetic control of micronutrients (Govindaraj et al., 2019).

A large variability for Fe and Zn content in germplasm and breeding lines has been indicated (Table 1), suggesting the feasibility of genetic enhancement for these micronutrients. The highest Fe and Zn have been found in germplasm accessions and mapping populations derived from the *iniadi* landrace (Velu et al., 2008; Govindaraj et al., 2016). Screening of more than 120 Indian commercial hybrids has shown 46–56 ppm Fe and 37–44 ppm Zn (Rai et al., 2016). To initiate the mainstreaming of Fe and Zn. in pearl millet, the levels of 42 ppm Fe and 32 ppm Zn have been set as a baseline in the Indian national testing and cultivar release policy in 2018 (AICPMIP, 2018). The daily recommended allowances for Indian adults are 17–21 and 10–12 mg/d for Fe and Zn, respectively.

**Table 1:** Genetic variability for grain iron and zinc content in germplasm, inbreds, commercial cultivars, and mapping populations of pearl millet.

**Genetic materials**	**No of genotypes**	**Fe (ppm)**	**Zn (ppm)**	**References**
Germplasm	191	51–121	46–87	Rai et al., 2014
Inbreds	45	34–102	34–84	Govindaraj et al., 2013
	28	30–82	27–56	Kanatti et al., 2014
	281	35–116	21–80	Pujar et al., 2020
Commercial hybrids	52	47–85	36–70	Velu et al., 2008
	120	46–56	37–44	
Populations (OPVs)	68	42–80	27–50	Velu et al., 2008
	18	42–67	37–52	
Population progenies	240	29–89	32–71	Govindaraj et al., 2016
	299	31–143	35–82	Govindaraj et al., 2019
Mapping populations	317	23–154	19–121	Mahendrakar et al., 2019
	106	28–124	29–120	Kumar et al., 2016

Fe and Zn contents in pearl millet are largely governed by additive genetic variance (Govindaraj et al., 2013; Kanatti et al., 2014), suggesting that both parental lines of hybrids would be required to improve for these micronutrients. Relatively lower G × E influences on the accumulation of Fe and Zn in pearl millet grains (Kanatti et al., 2014; Govindaraj et al., 2016) also indicated the effectiveness of progeny selection in the pedigree breeding to develop lines with increased levels of grain Fe and Zn densities. The higher additive genetic variance also prompts recurrent selection methods to be effective to improve the levels of grain Fe and Zn in breeding populations (Govindaraj et al., 2019).

A significant and positive association has been established between Fe and Zn (Govindaraj et al., 2013, 2016, 2020; Kanatti et al., 2014; Rai et al., 2014; Pujar et al., 2020). These two micronutrients also had a positive and highly significant correlation with seed size (Gupta et al., 2009; Kanatti et al., 2014; Govindaraj et al., 2016). Furthermore, gray and white grains from the same genetic background did not differ in their Fe/Zn levels (Govindaraj et al., 2018). These associations would give breeders leverages to develop Fe- and Zn-rich cultivars with large grain size irrespective of their color and to allow enhancement of micronutrients in mainstream breeding. The efforts in biofortification have yielded nutrient-rich and high-yield cultivars in India (Rai et al., 2014). Ten cultivars have been released with yield levels of 3.2–3.6 t/ha and 68–80 ppm Fe (Govindaraj et al., 2019), and several more are in the pipeline. Higher correlation between Fe and Zn in pearl millet exhibited that all these cultivars also had higher Zn levels (35–45 ppm Zn).

## Hybrid Replacement

Replacement of old hybrids by new ones with higher potential productivity is very critical in achieving continuous genetic gains. The design followed involves the identification of potential new hybrids well in advance through multiyear and multilocational testing in the National Coordinated Trials where their performance is judged against the best hybrid released most recently (AICPMIP, 2018). The strategy essentially involves keeping a close watch on the performance and disease incidence of hybrids in their production regions. There is ~15–20 public- and 30–40 private-sector organizations engaged in meeting the national annual demand of about 22,000 metric tons seed.

Once a hybrid passes through the research and development stage of 10–15 years involving its creation, evaluation, and registration, it is introduced in the market and goes through five stages of growth, maturity, saturation, decline, and replacement. To achieve sustainable growth in the seed business, a balanced product portfolio requires a minimum of one product at each stage from introduction to decline. The hybrids are introduced in different years and are generally phased out in a 10-year time frame. An average life cycle of 6 years of top five hybrids from introduction to retirement is maintained by different public and private organizations.

Although the hybrid life cycle is largely influenced by strong competition and breakdown of resistance to DM or blast, other factors, such as market demand, alternate product options, and product quality, also play an important role. In fact, the shorter life cycle due to competition has helped to an accelerated genetic gain due to the faster introduction of new hybrids with improved yield and resistance to diseases. This strategy has proved very critical to ensure a timely and adequate supply of desired hybrids to the farmers to sustain a continuous gain in productivity for the last few decades. With this strategy in place, ~60–70 hybrids are cultivated on the farm at any point of time in India for the last two decades.

## Realized Yield Gains

Pearl millet productivity has increased from 303 kg/ha during 1950–1954 to 1,239 kg/ha during 2015–2019 that translates to an increase of more than 300% owing to the widespread use of high-yielding and disease-resistant cultivars with improved production technology. A critical analysis of the genetic improvement has been recently done in which seven decades of breeding were divided in four phases, each phase having its uniqueness (Yadav et al., 2019). During phase I (1950–1966), when genetic improvement largely concentrated on the enhancement of yield in locally adapted materials, the rate of productivity improvement was 4.5 kg/ha per year. Discovery and utilization of CMS in hybrid development marked the second phase of genetic improvement (1967–1983) in which an annual increase of 6.6 kg/ha in productivity was realized despite the large-scale cultivation of a few hybrids. A large number of genetically diverse CMS lines were developed and utilized in hybrid breeding during phase III (1984–2000), and the productivity increase was 19.0 kg/ha per year. During phase IV (2001–2018) when genetic improvement put much greater emphasis on genetic diversification of hybrids and adaptation to niche areas of cultivation, the rate of improvement in grain productivity further increased to 31.1 kg/ha per year, which is 470% of the productivity gain achieved during the first phase.

**Figure 3:**
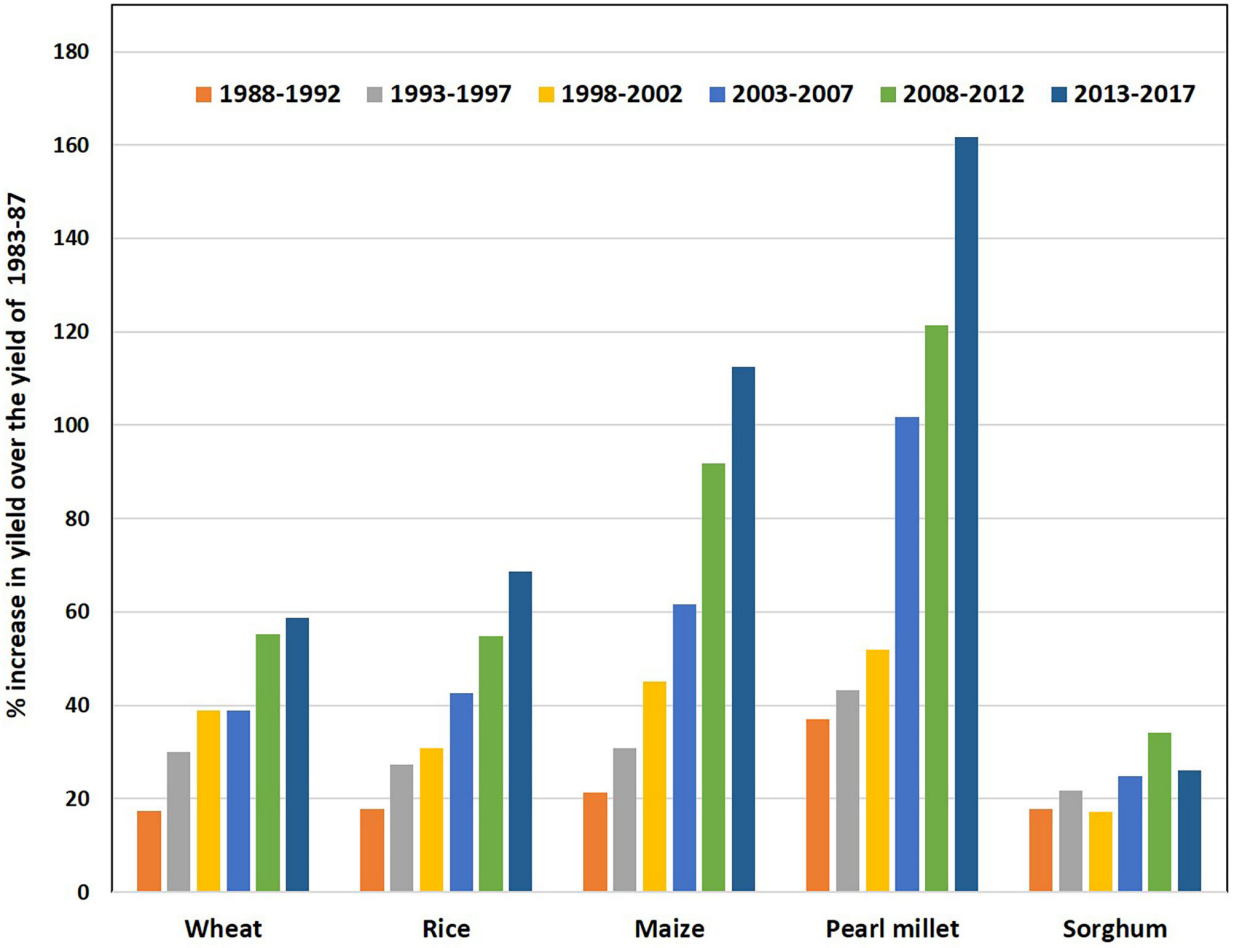
Per cent improvement in the national average yield of sorghum, wheat, rice, maize, and pearl millet from 1988 to 2017 over average yields of the quinquennial period of 1983-1987 (data source: www.agricoop.nic.in).

Comparison of yield increase in pearl millet vis-à-vis other four major cereals in India after the mid-1980s presents very interesting information. Following the adoption of high-yielding, disease-resistant, and stress-tolerant cultivars and crop management technology, there is a yield increase of 26% in sorghum, 59% in wheat, 69% in rice, 113% in maize, and 162% in pearl millet (Figure 3). These yield gains translate into 0.9% annual gains in sorghum, 2.0% in wheat, 2.3% in rice, 3.8% in maize, and 5.4% in pearl millet; all of these are much higher than the average gains achieved at the global level (FAO, 2020). The annual rate of gains in productivity is the combined outcome of improved genetics and management. This high quantum of productivity increase in pearl millet assumes greater significance in two ways. First, more than 90% of pearl millet is grown as rainfed and often on marginal lands. Second, pearl millet has attracted much lesser infrastructure and human resources in comparison to other food crops. It also affirms the correctness of priorities set in the breeding programs of India and simultaneously demonstrates the role of hybrid technology in raising crop productivity in marginal drylands.

## Prospects of Accelerating Genetic Gains

Pearl millet has shown impressive genetic gains in India for the past seven decades (Yadav et al., 2019). The crop now is poised to take the next quantum leap in genetic gains. The specific areas that are likely to contribute to the process are discussed in the following sections.

### Genomics-Assisted Breeding

One of the most exciting developments that have implications on taking the genetic gains to the next levels in pearl millet is genomics and genomics-assisted breeding that can help improve the precision and efficiency of the breeding program. The ~1,000 genomes sequencing project has been a major milestone in pearl millet improvement (Varshney et al., 2017). This work has laid a solid foundation for carrying out trait discovery, mapping, and deployment of QTLs/alleles/candidate genes linked to traits of economic interests. It also has helped toward the development and implementation of whole-genome prediction models for the pearl millet community globally (Jarquin et al., 2020; Srivastava et al., 2020a). The whole-genome resequencing of Pearl Millet Inbred Germplasm Association Panel, mapping population parents, and elite hybrid parental lines have helped to develop a huge (>32 million) repository of genome-wide SNPs. These developments offer opportunities to rapidly map and deploy genes of agronomic importance and also to rapidly resequence lines to mine and map genes of interest. The sequencing-based mapping strategy can also help us identify superior haplotypes for different traits to form the basis of haplotype-based breeding (Sinha et al., 2020).

Many traits of agronomic importance have been mapped related to diseases, terminal drought, grain and fodder quality, combining ability loci, and heterotic gene pools (Kumar et al., 2018; Basava et al., 2019; Gupta et al., 2020; Srivastava et al., 2020a,b) with SSR and SNP markers. The available QTLs can also be remapped using the currently available SNP-based high-throughput genotyping systems. This will allow integration into the modern breeding pipelines using high-throughput genotyping platforms available currently in pearl millet (Srivastava et al., 2020a,b). Furthermore, with the availability of reference genome sequence, large-scale whole-genome resequencing data, cost-effective genotyping platform, and precise phenotyping platforms (see later), it has become possible now to map breeding-related traits in a fast manner (Bohra et al., 2020). We believe the near future will be witnessing the deployment of genomic breeding approaches such as haplotype-based breeding, forward breeding, genomic prediction, and gene editing for pearl millet improvement (Varshney et al., 2020). Along with ICRISAT, a few Indian breeding centers are using markers for the selection of terminal drought tolerance and disease resistance. However, time to release has not been significantly impacted yet, as these technologies need to be integrated with speed breeding pipelines.

### Precision Phenotyping

While genotyping has become considerably cheaper and more precise in the recent past, precision phenotyping has been a major challenge, especially for drought. Full advantage of genomic resources can be taken only when quick, accurate, and cost-effective phenotypic data including root systems are available for genetic dissection of drought tolerance and selection of drought-resilient genotypes (Tuberosa, 2012). Usefulness of high-throughput and automated phenotyping platforms such as LeasyScan has been demonstrated in screening a large number of genotypes for drought tolerance (Vadez et al., 2015). There exists a much greater need to enhance the capacity for drought tolerance breeding programs to generate quick and accurate data through the use of drones, near-infrared imaging, and remote sensing.

### Heterotic Grouping of Hybrid Parental Lines

Heterotic grouping of hybrid parental lines is an important strategy to increase the magnitude of heterosis on a long-term basis (Melchinger and Gumber, 1998). A diverse range of breeding material has historically been used to develop either seed parents (B lines) or restorers parents (R lines), depending upon their specific phenotypic traits (Rai et al., 2006). Studies assessing molecular diversity classified such lines into genetically distinct groups and the confirmed existence of two broad-based groups in hybrid parents—one each for seed parents and restorer parents (Nepolean et al., 2012; Gupta et al., 2015a; Singh et al., 2018a).

Some attempts have been made in pearl millet to define heterotic groups. Pucher et al. (2016) investigated combining ability patterns in West African population hybrids but could not come out with clear heterotic groups probably because of genetic admixture in populations. Another study indicated seven heterotic groups among hybrid parents using EST and genomic SSR markers (Ramya et al., 2018). The existence of B and R lines as separate groups has been found responsible to behave as two separate broad heterotic pools, as B × R hybrids reported significantly higher levels of heterosis than B × B or R × R hybrids (Singh et al., 2018a). Recently, in a study involving 320 R and 260 B lines derived from pearl millet breeding programs in India, two each of B- and R-line heterotic groups were identified based on the heterotic performance and combining ability (Gupta et al., 2020). Hybrids from these identified B × R heterotic groups showed grain yield heterosis of more than 10% over the best commercial hybrid checks. This study also indicated that distinct parental groups can be formed based on molecular markers, which can help in assigning hybrid parental lines into heterotic groups to develop high-yielding hybrids. Now, the work is underway to select the appropriate testers to categorize the new hybrid parental lines or new germplasm into heterotic groups to enhance the genetic gains in pearl millet.

Combining ability studies conducted in pearl millet have shown that either there was no negative correlation or there was a positive correlation between their *per se* performance and general combining ability (GCA). Thus, high general combiners are likely to occur in lines with high grain yield *per se* than in any other yield group. Pearl millet breeding programs in India have been advancing progenies based on performance *per se* of lines during trait-based pedigree breeding, although now there is a shift toward combining ability-based selection approach. Considering the seed production economy and the high probability of producing high-yielding hybrids from high GCA lines, it is prudent that seed parents should possess both high yield *per se* and GCA.

There is not much information available about the extent of specific combining ability (SCA) and GCA variances in the existing B- and R-gene pools of pearl millet. A recent study has shown high SCA:GCA ratio (about 2 times), indicating the predominance of SCA variance over GCA variance in pearl millet hybrid parents, which was different than many of the established maize hybrid breeding programs of the United States and Europe, where low SCA:GCA ratio was observed (Gupta et al., 2020). Considering this scenario in other crops, there is a need to investigate SCA and GCA variances in the existing B- and R-line heterotic pools of pearl millet to better understand the contribution of GCA and SCA variances toward heterosis.

### Harnessing Genetic Diversity

World collection of germplasm (>23,000 from 52 countries) including wild species provides a great resource to look for new sources of economic traits, disease resistance, abiotic stress tolerance, and better nutritional quality. A small fraction of germplasm has been utilized so far primarily due to the huge number of germplasm accessions and the presence of undesired traits in the unadapted genetic background. These twin problems have been largely circumvented. Development of core and minicore collections (Upadhyaya et al., 2011) is prompting breeders to use the desired germplasm in broadening the genetic base of commercial cultivars, which is very essential to reduce the chances of disease epidemics and to mitigate the effects of climate change. Very recently, >1,000 accessions of pearl millet have been sequenced as well (Varshney et al., 2017). This, in combination with genome–environment association, would help in exploring the genetic basis of adaptive traits such as drought and heat tolerance in germplasm as has been demonstrated in other crops (Frank et al., 2016; Cortés and Blair, 2018).

The accessibility of such molecular tools offers a greater opportunity to transfer specific targeted regions and to minimize the linkage drag from agronomically inferior-looking germplasm accessions. Recently, new sources of blast resistance and flowering-stage heat tolerance have been developed in cultivated pearl millet backgrounds using wild *P. glaucum* subsp. v*iolaceum* (Sharma S. et al., 2020). To harness the untapped diversity, traits-specific germplasm needs to be targeted, which requires detailed characterization for new and novel traits proposed by breeders and the crop product profiles in the near future.

### Addressing Host Resistance and Pathogen Virulence Together

The experience, so far, in resistance breeding for DM has indicated that most of the hybrids become susceptible in about 5–6 years of cultivation in the same area because of selection pressure in the pathogen, although there are some clear exceptions where hybrids have shown durable resistance. It would be useful to investigate resistance mechanisms operative in the parents of such hybrids to identify and deploy genes for durable DM resistance in high-yielding hybrids for the enhanced genetic gain. Continuous monitoring of virulence of pathogen populations through reaction on host differential is essential to identify resistance effective against new virulent pathotypes. Genome sequencing of pearl millet (Varshney et al., 2017) and pathogens of DM and blast (Nayaka et al., 2017; Prakash et al., 2019) will help in understanding the molecular basis of compatible/incompatible host × pathogen interaction and provide a greater opportunity to breeders and pathologists to control the diseases. With the availability of genomic tools, identification of QTLs determining resistance to particular disease, and demonstration of the success of marker-assisted backcrossing, it now appears possible to stack target QTLs in the parental lines of commercial hybrids having multiple resistance to various pathotypes of DM, blast, and rust to realize a greater genetic gain. It should be recognized that QTLs transferred through backcrossing are pathotype-specific that indicates vertical resistance of their nature. These impart relatively large phenotypic variance and hence may house major genes for disease resistance. These may, however, get defeated by the pathogen relatively quickly; hence, identification of QTLs responsible for horizontal resistance is underway.

### Strengthening Hybrid Breeding for Arid Regions

One of the key issues, often debated in past, has been the comparative advantage of hybrids or Open-pollinated varieties (OPVs) under severe drought conditions, given the reports that genetically heterogeneous OPVs might exploit population buffering mechanism (Bradshaw, 1965; Haussmann et al., 2000) to provide stable performance under unpredictable drought environments. A comprehensive study conducted in arid regions of India comparing 142 hybrids and 84 composites over 12 years in 94 environments for their performance reported that hybrids yielded significantly higher grain than composites with an overall superiority of 25% (Yadav et al., 2012a).

Other consideration, in cultivar adoption in the existing seed supply system of pearl millet, is the multiple sowings, especially in drought areas. Composites have an edge over hybrids as they are self-perpetuating, and harvested seed can be used to plant the next crop. However, such an option would come with a significant penalty at least for grain yield. Therefore, hybrids are likely to play a much greater role than composites in enhancing pearl millet productivity in drought-prone regions including SSA.

### Mainstreaming the Biofortification

Mainstreaming of Fe and Zn in pearl millet breeding needs to be implemented to achieve nutritional security in SA and SSA. Breeding for micronutrients and vitamins has been initiated by HarvestPlus, a CGIAR Challenge Program. In collaborations with national partners, ICRISAT has generated now enough database for Fe and Zn. Greater than 60 ppm for Fe and 40 ppm for Zn were targeted for breeding as a part of the ICRISAT product profile. This is a momentous step toward mainstreaming in elite breeding lines. The dissemination of these mainstreamed breeding lines and hybrid parents and their utilization (both public and private sector) would make biofortified hybrid development a regular activity to enhance genetic gains for the micronutrient traits in the long term.

This is very encouraging that no negative association has been reported between grain yield and micronutrients in pearl millet, suggesting the feasibility of combining high yield with a greater concentration of micronutrients. Efficacy studies have indicated bioavailability of 7% of Fe and 25% of Zn in pearl millet. Biofortified pearl millet may provide up to 80% of Fe and 100% of Zn daily requirements and would have a greater impact on human health.

### Improving Nutrient Use Efficiency

Although pearl millet is mainly cultivated on sandy and sandy-loam soils that are inherently low in their nitrogen (N) and phosphorous (P) contents, its adaptation to low nutrients is seldom addressed assuming that this issue can be easily addressed through the external use of fertilizers. Limited studies conducted on this aspect (Gemenet et al., 2015) have indicated the possibility of breeding nutrient-use-efficient (NUE) cultivars of pearl millet. Looking to soil degradation and water contamination due to the leaching of N in subsurface or groundwater, pearl millet can be an important source of native genes that confer adaptation to low nutrient conditions (Serba et al., 2020). There is a need for a systematic study to understand the relevant traits' priority and their magnitude of variability for NUE using core breeding materials including minicore collections available at GenBank (Pujarula et al., 2021).

### Synergizing Breeding and Agronomics

A sustained increase in pearl millet productivity requires the integration of suitable cultural practices in its diverse production environments for disease resistant and improved cultivars to achieve greater genetic gains. On-farm demonstrations of improved cultivars and production technologies have established that the pearl millet yields at farm levels can easily be enhanced by 20–25% by adopting suitable agrotechniques (Yadav et al., 2012a). Intensive management including higher planting density, irrigation scheduling, and recommended use of mineral fertilizers in better-endowed areas would play a critical role in harnessing the potential yield of improved cultivars. Widely spaced crop, integration of legumes in pearl millet–based cropping system to maintain soil fertility, and microdosing of nutrients are very important to further enhance productivity gains in drought-prone regions. Machine harvestable plant type and lodging resistance are the need of the hour in reducing cultivation cost and enhancing profitability.

### Speed Breeding and Big-Data Analytics

Genetic gains of any breeding program significantly depend upon the number of crop breeding cycles a program can undertake in a year. This varies in different breeding programs as per their local weather conditions. For instance, only one crop of pearl millet can be taken in north India, while two crops per year can be grown in western, central, and peninsular India. Under this current scenario, breeding a new crop cultivar takes about a decade or more, with 6 or 7 years spent in seasonal generational advancements to arrive at elite materials that go for testing and release. Now, new environmentally controlled facilities, known as “RapidGen,” have been developed, which will shorten the 6–7-year window significantly. When used with the full suite of breeding acceleration techniques, RapidGen can make it possible to take four crop cycles in a year (https://www.icrisat.org/first-public-research-facility-to-put-agriculture-on-fast-forward-launched-at-icrisat/). With such new facilities in place, we are now moving toward the new era of speed breeding in pearl millet where genetic gains are poised to take a further leap.

Over the past several decades, judicious use of data analytics in multilocational trials and quantitative genetics played a major role in achieving higher genetic gains in pearl millet. However, the present era is of ultrahigh-speed computing, crop simulations, big-data analytics, internet of things, artificial intelligence, and machine learning and must be exploited in pearl millet breeding for achieving better genetic gains. These high-throughput streams need to be decoded for pearl millet improvement by developing an appropriate digital data capture platform, breeding databases, modern quantitative genetics, and real-time analytics. All such information in databases will provide an opportunity to run complex queries and scenario analysis enabling researchers to focus on specialized research. One extremely computationally intensive data science intervention will be the use of quantitative genetics–based crop simulation algorithms to understand and optimize existing pearl millet breeding pipelines and take measures to refine them further.

### Building Partnership

Pearl millet research and development are a mandate of several national and international organizations with a common goal of making an impact on the communities cultivating pearl millet. With this shared goal, the partnership needs to be pursued systematically. Partnership with international and advanced research institutes has contributed to providing access to well-characterized genetic resources and developing genomic resources. Partnership with the private sector has been most critical in delivering the products of improved genetics to the farming community. One such successful existing example of public-private partnership (PPP) is the ICRISAT-Private Sector Pearl Millet Seed Companies Consortium, established in 2000 and which has an engagement of ~30 seed companies. This consortium has proved to be a very effective vehicle to quickly deliver research outputs of ICRISAT's breeding program (Gowda et al., 2009). Another successful example of PPP is the International Pearl millet Genome Consortium, where 65 scientists from 30 research institutions across the world came together and unraveled the sequence of the pearl millet genome (Varshney et al., 2017). Such partnerships hold the key in providing adequate resources and making advances in cutting-edge science technologies to realize a sustained growth in pearl millet productivity.

## Conclusion

Pearl millet is becoming an indispensable food crop that provides calories, nutrition, and livelihood security to the poor and marginal people living in the fragile ecosystem of arid and semiarid regions of SA and SSA. Pearl millet is a crop of choice because of its critical role in enhancing the resilience to climate change. The past breeding priorities and strategies have been able to deliver significant productivity growth realized in pearl millet. Greater use of hybrid technology, employing modern tools, wider interinstitutional, and intersectoral partnerships, and improved crop management practices would play a greater role to accomplish much higher genetic gains for yield and nutritional traits for growing populations in SA and SSA. The success would depend upon a deeper understanding of new germplasm, genome, and trait-specific genes for novel traits through an amalgamation of conventional and modern tools and rapid generation techniques in national and international pearl millet breeding programs.

## Author Contributions

OY conceived the idea of writing this review and prepared the draft of the manuscript. SG, MG, RS, RV, RKS, AR, and RM contributed and strengthened different sections included in the review. All authors read and approved the manuscript.

## Conflict of Interest

RM was employed by company SeedWorks International Pvt Ltd, Hyderabad, India. The remaining authors declare that the research was conducted in the absence of any commercial or financial relationships that could be construed as a potential conflict of interest.
